# Clinical Validation of a Primary Antibody Deficiency Screening Algorithm for Primary Care

**DOI:** 10.1007/s10875-023-01575-8

**Published:** 2023-09-16

**Authors:** Marianne A. Messelink, Paco M. J. Welsing, Giovanna Devercelli, Jan Willem N. Marsden, Helen L. Leavis

**Affiliations:** 1 Department of Rheumatology & Clinical Immunology, University Medical Center Utrecht, Utrecht, Netherlands; 2 Takeda Development Center Americas, Deerfield, IL USA

**Keywords:** Primary antibody deficiencies, primary care database, diagnostic delay, screening algorithm, validation study

## Abstract

**Purpose:**

The diagnostic delay of primary antibody deficiencies (PADs) is associated with increased morbidity, mortality, and healthcare costs. Therefore, a screening algorithm was previously developed for the early detection of patients at risk of PAD in primary care. We aimed to clinically validate and optimize the PAD screening algorithm by applying it to a primary care database in the Netherlands.

**Methods:**

The algorithm was applied to a data set of 61,172 electronic health records (EHRs). Four hundred high-scoring EHRs were screened for exclusion criteria, and remaining patients were invited for serum immunoglobulin analysis and referred if clinically necessary.

**Results:**

Of the 104 patients eligible for inclusion, 16 were referred by their general practitioner for suspected PAD, of whom 10 had a PAD diagnosis. In patients selected by the screening algorithm and included for laboratory analysis, prevalence of PAD was ~ 1:10 versus 1:1700–1:25,000 in the general population. To optimize efficiency of the screening process, we refitted the algorithm with the subset of high-risk patients, which improved the area under the curve–receiver operating characteristics curve value to 0.80 (95% confidence interval 0.63–0.97). We propose a two-step screening process, first applying the original algorithm to distinguish high-risk from low-risk patients, then applying the optimized algorithm to select high-risk patients for serum immunoglobulin analysis.

**Conclusion:**

Using the screening algorithm, we were able to identify 10 new PAD patients from a primary care population, thus reducing diagnostic delay. Future studies should address further validation in other populations and full cost-effectiveness analyses.

**Registration:**

Clinicaltrials.gov record number NCT05310604, first submitted 25 March 2022

**Supplementary Information:**

The online version contains supplementary material available at 10.1007/s10875-023-01575-8.

## Introduction

Primary antibody deficiencies (PADs) form the majority of primary immunodeficiencies (PIDs) and are characterized by an inability to produce a clinically effective antibody response [1, 2]. PADs represent a heterogeneous group of disorders such as common variable immunodeficiency (CVID), IgG subclass deficiency, and specific antibody deficiency (SpAD) [3]. The reported prevalence of PAD varies considerably from 1:1700 to 1:25,000, partly owing to the suspected large number of undiagnosed patients [4–6]. The clinical presentation encompasses a wide range of symptoms including increased susceptibility to respiratory and gastro-intestinal tract infections, auto-immunity, and an increased risk of certain malignancies [2, 6].

Owing to the heterogeneous presentation and low prevalence, diagnosis of PAD can be challenging. This is evident from the reported median delay in diagnosis of between 2 and 10 years, which has not improved substantially over the past five decades [7–12]. This diagnostic delay is associated with increased morbidity and mortality, as effective therapies are available [12–14]. A timely diagnosis may also result in substantial healthcare cost savings, even when taking the cost of treatment into consideration [15]. Reducing the diagnostic delay of PAD is thus of key importance [12].

To this end, we have developed an algorithm that can be used to detect patients with a high risk of PAD in a primary care setting [16]. An advantage of focusing on primary care is that most patients initially present their complaints to a general practitioner (GP), especially in countries where the GP has a gatekeeper function to secondary care. This allows detection of PAD patients in an early phase. In addition, primary care electronic health records (EHRs) encompass a comprehensive overview of the symptoms for which a patient has sought medical care. In contrast, in secondary care, usually, only the symptoms for which a patient has been referred are documented structurally. For example, if a patient is referred for suspected inflammatory bowel disease, the secondary care EHR might not include recurrent respiratory tract infections for which a patient has visited the GP. Focusing on primary care thus allows screening for a broad range of PAD symptoms at an early stage.

The algorithm is based on structured EHR data including diagnostic codes, antibiotic prescriptions, laboratory results, and the number of visits to the GP. Focusing on structured EHR data enables the application of the algorithm in an automated manner to large databases. The aim of this study was to clinically validate and optimize the algorithm by applying it to a primary care database. Patients identified by the algorithm as being at increased risk of PAD were invited for laboratory evaluation of immunoglobulin levels and referred to an immunologist if deemed clinically necessary.

## Methods

Details on the algorithm have been reported previously and in Table S1 [16]. In short, the algorithm was developed using EHR data from PAD patients (University Medical Centre Utrecht), aggregated subgroup data from control groups (Julius General Practitioner Network (JHN) Utrecht), literature, and clinical expertise [17]. The algorithm encompasses 107 items within eight categories: “Antibiotic prescriptions,” “Respiratory tract infections” (RTI), “Gastro-intestinal (GI) complaints,” “Other infections,” “Auto-immune symptoms,” “Malignancies, lymphoproliferative- and other symptoms,” “Laboratory values,” and “Number of visits to the GP.”

In the current study, the algorithm was applied to a JHN data set containing 61,172 EHRs from 13 general practices. All patients in the JHN database were offered an opt-out prior to registration. EHR data were extracted for a certain period before the “censoring date” (e.g., 4 years for antibiotics; Table S1). Usually, this was the date of application of the algorithm to the database (8 February 2022). For certain uncommon diagnoses that can be both a complication of PAD and also the cause of a secondary antibody deficiency (SAD; e.g., non-Hodgkin lymphoma), the censoring date was the registration date of this ambiguous diagnosis (Table S1).

**Fig. 1:**
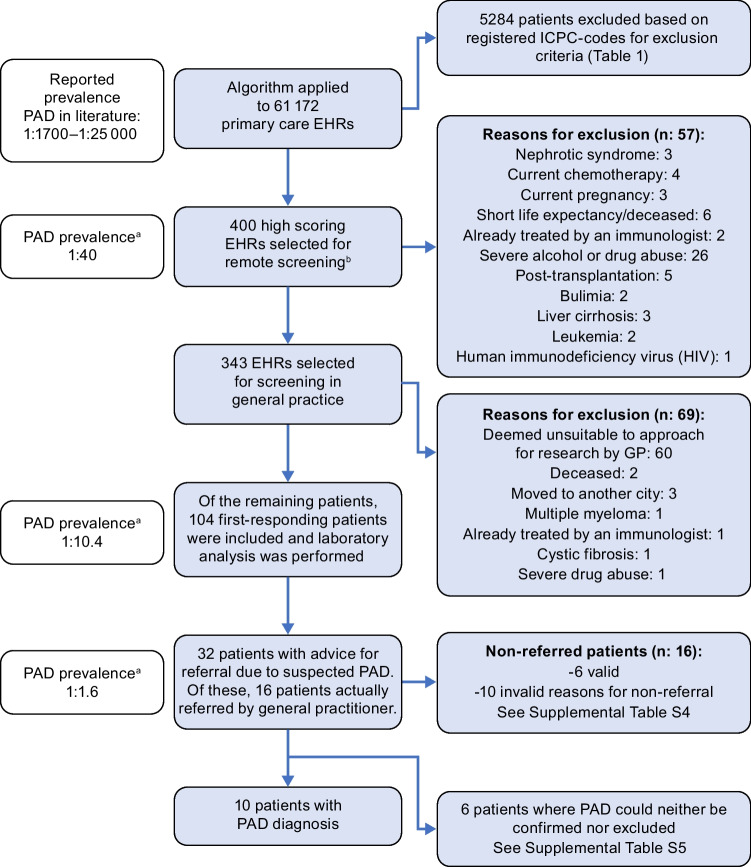
Overview of study flow. EHR electronic health record, GP general practitioner, ICPC International Classification of Primary Care, JHN Julius General Practitioner Network, PAD primary antibody deficiency. ^a^Prevalence was determined based on the 10 PAD patients in this data set. ^b^This selection included patients with a rank number < 400 due to an error in data extraction (see the “Results” section and Table S6)

As the estimated prevalence of PAD is 1:1700–1:25,000, 2–36 PAD cases were a priori expected to be present in the data set of 61,172 patients [4–6]. Previous PID-screening studies selected 0.1–0.4% of their population for further analysis [18, 19]. Based on the above, expert opinion, and feasibility, we aimed to screen the 400 highest scoring EHRs to confirm eligibility. Of the remaining patients, we aimed to include at least 100 patients (0.2% of the data set) for laboratory analysis. See Fig. 1 for an overview of the study flow. We focused on patients aged 12–70 years because PAD usually presents in the second to fourth decade of life, because of restrictions regarding study participation of patients < 12 years, and because differing clinical presentations have been described for children versus adults [8, 20–23].

Exclusion criteria (Table 1) for which International Classification of Primary Care (ICPC) codes were available were applied by the algorithm to the population of 61,172 patients and include previously diagnosed (secondary) immunodeficiencies. Subsequently, exclusion criteria were verified manually in the 400 EHRs selected for screening. This was performed in a two-step manner owing to COVID-19 restrictions. First, pseudonymized EHRs were screened remotely from a secured server, and subsequently, remaining patients were discussed with the GP on location.

**Table 1:** Exclusion criteria

Leukaemia (B73)	
Multiple myeloma (B74.01)	
HIV (B90, B90.01, B90.02)	
Anorexia nervosa / bulimia (T06, T06.01, T06.02)	
Cystic fibrosis (T99.10)	
Severe alcohol addiction (P15.01, P15.02, P15.03)	
Addiction to hard drugs (P19.03)	
Previously diagnosed immunodeficiency (T99.01)	
Nephrotic syndrome	
Stadium 3–4 liver cirrhosis	
Current systemic chemotherapy	
Current pregnancy	
Short life expectancy/deceased	
Patient is already treated by an immunologist	
Patient is not deemed suitable for participation by GP	

*EHR* electronic health record, *GP* general practitioner, *HIV* human immunodeficiency virus, *ICPC* International Classification of Primary Care

If an ICPC code was available, this is represented in the table in brackets. Exclusion criteria were applied by the algorithm if an ICPC code was available and subsequently verified by manually screening the EHR of the 400 highest scoring patients

Patients that remained eligible after screening were invited for participation through a letter from their GP. Participation consisted of a single visit with analysis of serum immunoglobulins and calculated globulin, and the Early Warning Signs (EWS) questionnaire [24, 25]. GPs were advised to refer patients with reduced immunoglobulins for further evaluation of PAD to an immunologist or infectious disease specialist, except if it concerned solitary reduced IgG4 as this has little clinical relevance [26]. In addition, GPs were advised to refer the 10% highest scoring patients, to account for SpAD which presents without concomitant reduced immunoglobulins [27]. Lastly, GPs were advised to consult an internist in case of incidental findings of elevated immunoglobulins that were not suspect for PAD. Six months after inclusion, GPs were contacted to verify referral outcomes. PAD was classified according to the International Union of Immunological Societies criteria by a clinical immunologist [28]. This study was approved by the Medical Research Ethics Committee NedMec under protocol number NL74 944.041.20. All patients included for laboratory analysis provided written informed consent. For the TRIPOD checklist, see Table S2.

Descriptive statistics are presented as means with standard deviations, medians with interquartile ranges, or frequencies with percentages. To compare continuous data between PAD and non-PAD patients, *t*-tests or non-parametric Wilcoxon rank sum tests (for two groups) were performed or for two groups or more ANOVA or Kruskal-Wallis (non-parametric) tests. For categorical characteristics, chi-square tests were used, or Fisher’s exact test if small cell frequencies were expected (< 5).

In addition to the original algorithm (version 1), we explored three alternative algorithms (versions 2–4) to optimize predictive performance within the subset of high-risk patients with a confirmed PAD/non-PAD diagnosis. We performed penalized logistic regression analyses, with the presence of PAD as a dependent variable. In the original algorithm (version 1), the eight categories were weighted equally. In version 2, category weights were adjusted based on Ridge regression coefficients (*λ* = one standard error), with the category scores as independent variables. In version 3, the items (e.g., “pneumonia”) per category (e.g., “RTI”) were first grouped using a principal component (PC) analysis to prevent overfitting due to the large number of items compared with the number of patients. The number of PCs was based on an eigenvalue of ≥ 1. Items were grouped in the PC where they had the highest contribution, or based on clinical rationale if they were not present in this data set. Version 3 was derived by first determining the weight per item group, and subsequently the weight per category using ridge regression. In version 4, we explored the addition of new variables that were not available during algorithm development or have an ambiguous relationship to PAD, i.e., use of immunosuppressant medication in the past 4 years, presence of an ICPC code for chronic obstructive pulmonary disease or malignancy, ≥ 6 GP visits in the past 2–4 years, and the EWS score. These variables and the total score of the optimal algorithm (from versions 1–3) were combined in a Lasso regression. Algorithm 4 consisted of the retained variables. The predictive performance of all algorithms was determined with the area under the curve–receiver operating characteristics curve (AUC-ROC), sensitivity, and specificity using optimal cut-offs based on Youden’s index or 100% sensitivity. Statistics were performed in R version 4.2.0 (The R Foundation for Statistical Computing, Vienna, Austria).

## Results

The algorithm was applied to 61,172 EHRs; 5284 patients were excluded based on ICPC codes (see Table 1) of which 8 had a previous PAD diagnosis. From the remaining 55,888 patients, 400 high-ranking patients were selected for EHR screening. Of these 400 patients, 104 were included for immunoglobulin assessment. From these, 16 were referred, of whom 10 were diagnosed with a PAD (Table S3). Sixteen patients were not referred despite referral advice, of which 6 were deemed valid and 10 invalid (Table S4). For example, a sufficient explanation for frequent antibiotic use was deemed a valid reason, while “referral was too much effort” was deemed invalid, as PAD cannot be excluded in this case. The valid non-referred patients (*n* = 6) and the patients without referral advice (*n* = 72) were labelled as “unlikely PAD diagnosis” (*n* = 78). For 6 referred patients PAD could not be confirmed nor excluded (Table S5); together with the invalid non-referred patients (*n* = 10), these were labelled as “inconclusive” (*n* = 16). The prevalence of PAD in the subpopulations selected with each step of the study is shown in Fig. 1. In the general population, the prevalence of PAD is estimated to be 1:1700–1:25,000 [4–6]. In the 400 patients selected for EHR screening, prevalence was estimated at 1:40, patients in whom immunoglobulin analyses were performed at ~ 1:10, and in those referred for suspected PAD at ~ 1:2. This can be translated to a number needed to screen of 40, a number needed to test of 10, and a number needed to refer of 2 to identify one patient with PAD.

Initially, we aimed to select the 400 highest scoring EHRs for screening. After termination of the study, it appeared, however, that lower ranks were also screened owing to a data-extraction error concerning antibiotic prescriptions, about which the ethical committee was informed (see Table S6 for details). The included patients were still within the highest scoring 2% of the total population of 61,172 patients (Figure S1). An unintended benefit of this occurrence is that it allowed us to study patients with a wider range of ranks. Three of the newly identified PAD patients had a rank lower than 400 (528, 657, and 791), as well as one patient with an inconclusive diagnosis (803). It thus appears that the initially estimated cut-off point of 21.5 (based on 400 highest ranks) was too strict, a finding which would have remained undetected if we had only screened the top 400 EHRs. A cut-off of ≥ 17 (corresponding to a rank of 1000) may be more suitable, as all confirmed and inconclusive PAD cases are well within this range.

The baseline characteristics are shown in Table 2; there were no statistically significant differences between groups of included patients (i.e., “PAD diagnosis,” “unlikely PAD,” and “inconclusive diagnosis”). The algorithm scores are shown in Table 3. Statistically significant differences were present for the total score and for the categories “Antibiotic prescriptions” and “RTIs,” but not for other categories. Most points were scored in the categories “Antibiotic prescriptions,” “RTIs,” and “Visits to the GP,” while points were rarely scored for “Other infections” (e.g., meningitis, osteomyelitis; Table S1) and “Auto-immune symptoms.” There were no previously registered reduced immunoglobulin levels in the EHRs of included patients, most likely because these were not requested by GPs: total IgG and IgM were determined in only one patient, and IgG subclasses in none.

**Table 2:** Baseline characteristics of included and screened patients

	Included patients (*n* = 104)			
	PAD diagnosis	Unlikely PAD^a^	Inconclusive^b^	Screened patients
*n*	10	78	16	400
Age (y) mean (SD)	58.4 (6.4)	51.9 (14.8)	55.8 (11.8)	52.3 (14.9)
Patients aged 12–18 years *n* (%)	0 (0%)	4 (5.1%)	0 (0.0%)	18 (4.5%)
Patients aged 60–70 years *n* (%)	4 (40.0%)	31 (39.7%)	8 (50.0%)	168 (42.0%)
Female *n* (%)	8 (80.0%)	67 (85.9%)	14 (87.5%)	294 (73.5%)
Caucasian *n* (%)	9 (90.0%)	62 (79.5%)	13 (18.3%)	NA
Pack years median (IQR)	22.5 (22.5–24.0)	11.3 (2.9–24.0)	22.0 (12.0–24.0)	NA
JMF 10 Early Warning Signs, median (IQR)	3.5 (2.3–4.0)	2.0 (1.0–4.0)	3.0 (2.0–4.0)	NA

*IQR* interquartile range, *JMF* Jeffrey Modell Foundation, *n* number, *NA* not applicable, *PAD* primary antibody deficiency, *SD* standard deviation

There were no statistically significant differences among groups of included patients

^a^Included patients who did not receive an advice for referral for suspected PAD or who were not referred by their GP based on valid reasons (Table S4). Referral was advised based on reduced immunoglobulin results or if patients were within the top 10% of highest scoring patients on the algorithm

^b^Diagnosis was inconclusive if (1) referral was advised, but the patient was not referred based on invalid reasons (Table S4) or (2) the patient was referred, but PAD could not be confirmed nor excluded (see Table S5)

**Table 3:** Algorithm score, total and per category

	Included patients (*n* = 104)				
	PAD diagnosis (*n* = 10)	Unlikely PAD^a^ (*n* = 78)	Inconclusive^b^ (*n* = 16)	Sig.	Screened patients (*n* = 400)
Total score on algorithm median (IQR)	24.8 (20.8–31.5)	22.0 (19.0–29.0)	29.8 (25.1–44.3)	*	22.0 (19.0–29.0)
Antibiotic prescriptions					
Number of prescriptions in 4 years, median (IQR) (range)	5.0 (2.0–13.8) (2.0–25.0)	4.0 (2.0–7.0) (0.0–17.0)	9.5 (6.0–15.0) (4.0–31.0)	*	4.0 (2.0–7.0) (0.0–45.0)
Score, median (IQR)	7.3 (3.3–17.0)	6.8 (4.0–14.0)	16.9 (10.4–28.0)	*	7.0 (3.0–14.0)
RTIs					
Number of ICPC codes, median (IQR) (range)	5.5 (5.0–6.0) (3.0–10.0)	5.0 (4.0–6.0) (0.0–15.0)	4.0 (2.8–5.3) (1.0–6.0)	*	5.0 (4.0–6.0) (0.0–15.0)
Score, median (IQR)	9.5 (7.5–11.8)	9.0 (7.0–11.8)	6.0 (5.0–9.3)	*	9.0 (6.8–11.0)
GI complaints					
Number of ICPC codes, median (IQR) (range)	1.0 (0.3–1.8) (0.0–2.0)	1.0 (0.0–2.0) (0.0–3.0)	0.5 (0.0–2.0) (0.0–2.0)		1.0 (0.0–1.0) (0.0–4.0)
Score, median (IQR)	2.0 (0.3–2.8)	2.0 (0.0–3.0)	0.5 (0.0–2.3)		2.0 (0.0–2.0)
Other infections^c^					
Number of ICPC codes, median (IQR) (range)	0.0 (0.0–0.0) (0.0–0.0)	0.0 (0.0–0.0) (0.0–0.0)	0.0 (0.0–0.0) (0.0–1.0)		0.0 (0.0–0.0) (0.0–2.0)
Score, median (IQR)	0.0 (0.0–0.0)	0.0 (0.0–0.0)	0.0 (0.0–0.0)		0.0 (0.0–0.0)
Auto-immune symptoms					
Number of ICPC codes, median (IQR) (range)	0.0 (0.0–0.0) (0.0–2.0)	0.0 (0.0–0.0) (0.0–3.0)	0.0 (0.0–0.0) (0.0–3.0)		0.0 (0.0–1.0) (0.0–3.0)
Score, median (IQR)	0.0 (0.0–0.0)	0.0 (0.0–0.0)	0.0 (0.0–0.0)		0.0 (0.0–1.0)
Malignancy/lymphoproliferative/other symptoms					
Number of ICPC codes, median (IQR) (range)	1.0 (1.0–1.0) (0.0–1.0)	1.0 (0.0–1.0) (0.0–3.0)	1.0 (1.0–1.0) (0.0–2.0)		1.0 (0.0–1.0) (0.0–5.0)
Score, median (IQR)	1.0 (1.0–1.0)	1.0 (0.0–1.0)	1.0 (1.0–1.3)		1.0 (0.0–1.1)
Previously registered reduced immunoglobulin levels in EHR					
Number of patients (%)	0 (0.0%)	0 (0.0%)	0 (0.0%)		2.0 (0.5%)
Score, median (IQR)	0.0 (0.0–0.0)	0.0 (0.0–0.0)	0.0 (0.0–0.0)		0.0 (0.0–0.0)
≥ 6 visits to the GP per year					
Total number of visits, median (IQR) (range)	15.5 (10.3–24.8) (6.0–40.0)	16.0 (10.0–21.0) (3.0–21.0)	11.5 (8.0–19.3) (2.0–58.0)		15.0 (10.0–24.0) (2.0–61.0)
Score, median (IQR)	3.0 (3.0–3.0)	3.0 (3.0–3.0)	3.0 (3.0–3.0)		3.0 (3.0–3.0)

*EHR* electronic health record, *GI* gastro-intestinal, *GP* general practitioner, *ICPC* International Classification of Primary Care, *IQR* interquartile range, *n* number, *PAD* primary antibody deficiency, *RTI* respiratory tract infection, *Sig* significance

Score per category of the algorithm is based on the presence of items (e.g., ICPC code for pneumonia) multiplied by the weight of that item (see Table S1). *Statistically significant difference among groups of included patients

^a^Included patients who did not receive an advice for referral for suspected PAD or who were not referred by their GP based on valid reasons (Table S4). Referral was advised based on reduced immunoglobulin results or if patients were within the top 10% of highest scoring patients on the algorithm

^b^Diagnosis was inconclusive if (1) referral was advised, but the patient was not referred based on invalid reasons (Table S4) or (2) the patient was referred, but no definite diagnosis could be made (see Table S5)

^c^See Table S1 for the diagnostic codes that belong to this category, which includes, e.g., meningitis and septic arthritis

The serum immunoglobulin results are shown in Table 4. PAD patients had significantly lower IgM, total IgG, and IgG1, IgG2, and IgG3 subclass levels than “unlikely PAD” patients. There were no statistical differences for IgA nor IgG4 subclass levels. Concerning calculated globulin, PAD patients had a significantly lower median value, but none of the patients had a value below the diagnostic cut-off of 18 g/L [25].

**Table 4:** Serum measurements of immunoglobulins (gram/liter)

	PAD diagnosis (*n* = 10)	Unlikely PAD^a^ (*n* = 78)	Inconclusive ^b^ (*n* = 16)	Sig.
IgM median (IQR)	0.52 (0.40–0.87)	0.98 (0.74–1.38)	0.77 (0.43–1.22)	*/**
IgM *n* (%) reduced	3 (30.0%)	5 (6.4%)	4 (25.0%)	*/**
IgA median (IQR)	2.07 (1.52–2.32)	2.09 (1.38–2.84)	2.20 (1.68–2.97)	
IgA *n* (%) reduced	0 (0.0%)	2 (2.6%)	0 (0.0%)	
IgG total median (IQR)	7.28 (6.12–8.52)	9.89 (8.24–11.18)	9.77 (8.73–11.38)	*/**
IgG total *n* (%) reduced	5 (50.0%)	3 (3.8%)	1 (6.3%)	*/**
IgG1 median (IQR)	5.00 (4.43–6.90)	6.80 (5.90–7.78)	6.75 (6.05–7.85)	*/**
IgG1 *n* (%) reduced	5 (50.0%)	4 (5.1%)	2 (12.5%)	*/**
IgG2 median (IQR)	1.61 (1.18–2.08)	2.60 (1.90–3.20)	2.40 (1.43–2.98)	*/**
IgG2 *n* (%) reduced	5 (50.0%)	4 (5.1%)	5(31.3%)	*/**
IgG3 median (IQR)	0.22 (0.16–0.28)	0.22 (0.16–0.28)	0.38 (0.32–0.49)	*/**
IgG3 *n* (%) reduced	4 (40.0%)	5 (6.5%)	4(25.0%)	*/**
IgG4 median (IQR)	0.24 (0.15–0.34)	0.33 (0.14–0.52)	0.50 (0.30–1.04)	
IgG4 *n* (%) reduced	1 (10.0%)	9 (11.5%)	2 (12.5%)	
Calculated globulin median (IQR)	27.0 (25.0–28.0)	30.0 (28.0–33.0)	32.0 (28.8–34.3)	*/**
Calculated globulin *n* (%) reduced	0 (0.0%)	0 (0.0%)	0 (0.0%)	

*Ig* immunoglobulin, *IQR* interquartile range, *PAD* primary antibody deficiency, *Sig.* significance, *yrs* years

Values expressed as gram/liter. *Overall statistically significant difference among the three groups of patients; **statistically significant difference between “PAD diagnosis” and “Unlikely PAD” groups

^a^Included patients who did not receive an advice for referral for suspected PAD or who were not referred by their GP based on valid reasons (Table S4). Referral was advised based on reduced immunoglobulin results or if patients were within the top 10% of highest scoring patients on the algorithm

^b^Diagnosis was inconclusive if (1) referral was advised, but the patient was not referred based on invalid reasons (Table S4) or (2) the patient was referred, but no definite diagnosis could be made (see Table S5). Values were considered reduced when IgM < 0.4 (< 0.28 age 12–16 years), IgA < 0.7, IgG total < 7 (< 5.2 age 12–16 years), IgG1 < 4.9 (< 3.7 age 12–16 years), IgG2 < 1.5 (< 1.06 age 12–18 years), IgG3 < 0.20 (< 0.18 age 12–18 years), IgG4 < 0.08 (< 0.035 age 12–18 years), and calculated globulin < 18

To optimize the efficiency of the screening approach, we aimed to improve the predictive performance within the subset of high-risk patients identified by the original algorithm, i.e., the 88 confirmed PAD/unlikely PAD cases (see Table 5). When considering the total data set of 61,172 patients with 10 new PAD patients and all others assumed to be free of PAD, the original algorithm has an estimated AUC-ROC of 0.99 (95% confidence interval (CI) 0.99–0.99). However, when the original algorithm (version 1) is applied to the subset of 104 high-risk patients, the AUC-ROC is 0.58 (95% CI 0.39–0.78). In version 2 of the algorithm, category weights were adjusted as follows based on the ridge regression coefficients: “GI-complaints” was reduced to 0.5; “Antibiotic prescriptions,” “RTIs,” and “Malignancy, lymphoproliferative- and other symptoms” remained as 1; and “Auto-immune symptoms”, “GP-visits,” and “Other infections” were increased to 2. This did not improve algorithm performance; the AUC-ROC remained as 0.58. The weights per item group and per category in version 3 of the algorithm, based on the ridge regression coefficients, are shown in Table S7. This version improved the AUC-ROC to 0.80. In version 4, only the variables “algorithm score version 3” and “EWS score” were retained, but this did not improve the AUC compared with version 3 (AUC-ROC 0.80).

**Table 5:** Predictive performance of different versions of the algorithm

	Version 1	Version 2	Version 3^b^	Version 4^b^
Estimated^a^ AUC-ROC in total population of 61,172 patients	0.99 (95% CI 0.99–0.99)	-	-	-
Within subset of high-risk patients*				
AUC-ROC	0.58 (95% CI 0.39–0.78)	0.58 (95% CI 0.38–0.79)	0.80 (95% CI 0.63–0.97)	0.80 (95% CI 0.63–0.97)
Optimal cut-off based on Youden’s index	23.0 Sensitivity, 70% Specificity, 50%	21.0 Sensitivity, 70% Specificity, 53%	15.5 Sensitivity, 90% Specificity, 68%	16.2 Sensitivity, 90% Specificity, 69%
Optimal cut-off based on 100% sensitivity	18.0 Specificity, 8%	16.0 Specificity, 12%	9.5 Specificity, 9%	9.6 Specificity, 9%

*1SE* one standard error, *AUC-ROC* area under the receiver-operator curve, *CI* confidence interval, *GI* general practitioner

^a^When considering the total data set of 61,172 patients with 10 new PAD patients identified and all others assumed to be free of disease

^b^For versions 3 and 4 of the algorithm, a principal component analysis was performed, using all available individual patient data from the Julius General Practitioner Network (*n* = 580). For version 4, a Lasso regression analysis was performed with *λ* = minimal *λ*, as no variables were retained in the model with *λ* = 1SE

*Eighty-eight confirmed PAD/unlikely PAD cases (i.e., 104 included minus 16 inconclusive patients)

**Fig. 2:**
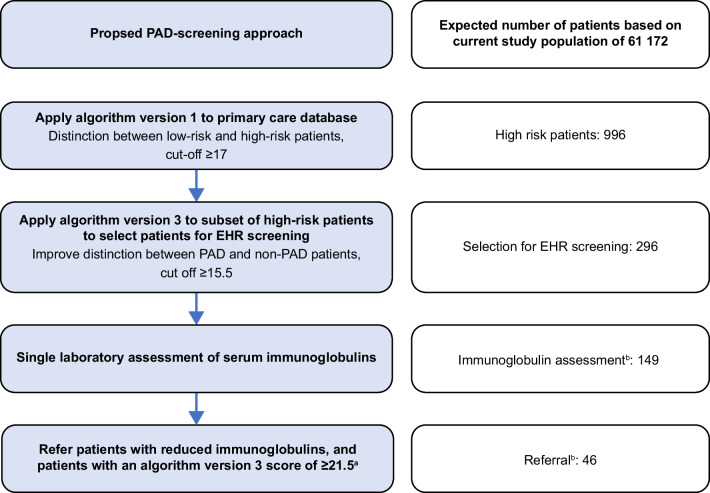
EHR electronic health record, PAD primary antibody deficiency, SpAD specific antibody deficiency. ^a^Reduced immunoglobulins, with the exception of isolated reduced IgG4 as this has little clinical relevance[26]. Refer patients with a high algorithm version 3 score of ≥ 21.5 (specificity~90%) to account for SpAD. ^b^Based on extrapolation from our current study data using multiple imputation

Based on these results, we suggest a two-step PAD screening approach using algorithm versions 1 and 3 (see Fig. 2). First, version 1 can be applied to a primary care database to distinguish low-risk from high-risk patients using a cut-off of ≥ 17. This cut-off is based on the lowest score of the identified PAD and inconclusive patients of 18, maintaining a safety margin of 1 point. Within this subset of high-risk patients, algorithm version 3 can be applied to improve the distinction between PAD and non-PAD patients using a cut-off of ≥ 15.5 based on Youden’s index (sensitivity 90%, specificity 68%). A cut-off of ≥ 9.5 points based on a 100% sensitivity may also be considered, although this greatly reduced the specificity to 9%. For the remaining patients, the EHR should be screened to confirm the absence of exclusion criteria. Patients satisfying inclusion/exclusion criteria can then be invited for immunoglobulin analysis by their GP. Referral to an immunologist can be advised for patients with reduced immunoglobulins with the exception of isolated reduced IgG4 as this has little clinical relevance [26]. Referral can also be advised for patients with a high algorithm version 3 score of ≥ 21.5 (based on specificity ~ 90%) to account for SpAD, as this can present without reduced immunoglobulin levels.

We estimated the costs for the proposed screening approach based on our population of 61,172 patients. See Fig. 2 for the expected numbers of patients per step of the proposed screening approach. The total estimated cost for this screening approach is €52,586.68, assuming that all invited patients participate. These costs include manual EHR screening for 296 patients, immunoglobulin assessment for 149 patients, and two visits to an academic hospital including additional laboratory assessments for 46 patients (see Table S8 for details). Based on the 10 patients we identified in this study, the estimated cost per detected patient is €5258.66. Initial costs for development of a certified digital screening tool were not taken into account. Potentially, the costs for EHR screening could be reduced by automating the process using text-mining techniques.

## Discussion

In the current study, we aimed to detect PAD patients using a screening algorithm in primary care. From a population of 61 172 patients, we included 104 high-risk patients for laboratory analysis, of whom finally 10 PAD patients were identified. A priori, we expected 2–36 PAD patients to be present in our total study population of 61,172, based on the prevalence of PAD in the general population [4–6]. As we identified 18 PAD patients in total (i.e., 10 new diagnoses and eight previous diagnoses), this is well within the range of expected PAD patients. Our original screening algorithm (version 1) performed well when distinguishing low-risk from high-risk patients, as the PAD prevalence in included patients was 1:10, compared with 1:1700–1:25,000 in the general population. Within the subset of high-risk patients, the original algorithm did not perform well (AUC-ROC 0.58), but predictive performance improved with an optimized version of the algorithm (AUC-ROC 0.80). We therefore propose a screening approach combining these two algorithms. To our knowledge, this is the first study to identify new PAD patients within a primary care adolescent/adult population using a screening algorithm.

The 10 PAD patients identified in this study had either an isolated IgG subclass deficiency or SpAD. Similar to Rider et al., we did not encounter selective IgA deficiency, which is a more prevalent but also milder type of PAD [29]. Possibly IgA-deficient patients were not classified as high risk because the majority are asymptomatic [30]. We also did not encounter more severe diagnoses such as CVID, which may explain the absence of patients with reduced calculated globulin levels [25, 31]. As CVID has a relatively low prevalence, cases may be encountered when applying the algorithm to a larger population. In addition, PAD cannot be excluded for high-risk patients who did not respond to the invitation from their GP nor for patients for whom referral advice was ignored. It is therefore possible that undetected (more severe) PAD cases are present in our included study population.

When designing the PAD screening algorithm, we were inherently limited by the available (diagnostic) codes in primary care, which tend to be more general (e.g., “Pneumonia”) rather than specific (e.g., “Mycoplasma Pneumonia”). Further limitations include that we initially intended to screen the 400 highest screening EHRs, but owing to a data-extraction error, lower ranking EHRs were also screened. An unintended benefit of this error was that it allowed for the study of patients with a wider range of algorithm scores. As three newly identified PAD patients had a rank lower than 400, we can conclude that our initially estimated cut-off point was too strict. This would have remained undetected if we had screened only the top ranking 400 patients. Lastly, as our optimized algorithm (version 3) was developed in a relatively small data set of high-risk patients, it should be validated in another population.

As for all screening methods, cost-effectiveness should be taken into account before considering implementation. Our initial rough estimate of the costs per detected PAD patient with our proposed screening approach is €5258. This is comparable to the costs per detected patient for other screening programmes in the Netherlands, such as for breast cancer (€9300), colon cancer (€5000), and cervical cancer (€8400) [32–34]. Of note, these screening programmes include more invasive interventions such as colonoscopy, compared with the laboratory assessment in our approach. The estimated annual cost savings for early PID diagnosis are $85,882 (≈ €81,157) for patients without immunoglobulin replacement therapy and $6500–$55,882 (≈ €6066–€52,158) for patients with this therapy [35–37]. When assuming that our approach would reduce the diagnostic delay of PAD by 3 years (median diagnostic delay 2–10 years [7–12]), this would imply a cost saving of €18,198–€243,471 over 3 years per detected PAD patient. This seems well proportionate to the expected cost per detected PAD patient of €5258. It is however important to note that the estimations for cost savings are based on PID patients in general rather than specifically PAD and that these studies were performed in other countries. A full cost-effectiveness analysis specific to our PAD screening approach would therefore be of interest.

The impact of participation in screening and a possible subsequent PAD diagnosis for individual patients should be taken into account. Our screening approach selects undiagnosed patients that have registered complaints associated with PAD. An early diagnosis could offer these patients an explanation for their complaints, and future complications may be prevented by starting adequate treatment. The benefits of an early diagnosis are thus likely to outweigh the burdens.

Other efforts to reduce the diagnostic delay of immunodeficiencies include the approaches developed by Rider et al. and Mayampurath et al., which could be complementary to the screening algorithm described in the current study [29, 38–40]. For example, these approaches focus mainly on secondary/tertiary care and use International Classification of Diseases diagnostic codes, while our approach specifically focuses on primary care and incorporates ICPC codes. Considering that diagnostic coding practices differ per country (ICPC being used in 27 countries), it is important to develop screening tools for both systems [41]. In addition, the approach by Rider et al. is based on paediatric data, while we specifically focus on PAD in an adolescent/adult primary care population aged 12–70 years. We chose to target this population as different cut-offs are to be expected in a paediatric population owing to the higher frequency of RTIs and because most PADs present between the second and fourth decade of life [8, 20–23]. X-linked agammaglobulinemia (XLA) is an exception, as this presents during the first few years of life. However, the diagnostic delay of XLA is limited (e.g., reported median of 1 year compared with 7.5 years for PAD in general [8]), and the proposed addition of XLA to newborn screening is likely a more effective diagnostic strategy for this particular PAD [42, 43].

In conclusion, in the current study, we were able to identify 10 new PAD patients from a primary care population of 61,172 patients using a PAD screening algorithm. We also present an optimized screening approach including a revised algorithm to improve predictive performance within high-risk patients. This approach may aid in the prevention of morbidity and mortality by reducing diagnostic delay of PAD and appears to be cost-effective based on a limited analysis. Future studies should address further validation of the proposed screening approach in other populations and a full cost-effectiveness analysis.

### Supplementary Information

Below is the link to the electronic supplementary material.

Supplementary file1 (DOCX 181 kb)

## Data Availability

Data are handled according to the FAIR principles. For access to the scripts for statistical analysis or study, data an application can submitted to the corresponding author, which will be reviewed by the study team.

## References

[1] Wood PM (2010). Primary antibody deficiency syndromes. Curr Opin Hematol.

[2] Durandy A, Kracker S, Fischer A (2013). Primary antibody deficiencies. Nat Rev Immunol.

[3] Fried AJ, Bonilla FA (2009). Pathogenesis, diagnosis, and management of primary antibody deficiencies and infections. Clin Microbiol Rev.

[4] Boyle JM, Buckley RH (2007). Population prevalence of diagnosed primary immunodeficiency diseases in the United States. J Clin Immunol.

[5] Bousfiha AA, Jeddane L, Ailal F, Benhsaien I, Mahlaoui N, Casanova JL (2013). Primary immunodeficiency diseases worldwide: more common than generally thought. J Clin Immunol.

[6] Wood P, Stanworth S, Burton J, Jones A, Peckham DG, Green T (2007). Recognition, clinical diagnosis and management of patients with primary antibody deficiencies: a systematic review. Clin Exp Immunol.

[7] Seymour B, Miles J, Haeney M (2005). Primary antibody deficiency and diagnostic delay. J Clin Pathol.

[8] Slade CA, Bosco JJ, Giang TB, Kruse E, Stirling RG, Cameron PU (2018). Delayed diagnosis and complications of predominantly antibody deficiencies in a cohort of Australian adults. Front Immunol.

[9] Aghamohammadi A, Bahrami A, Mamishi S, Mohammadi B, Abolhassani H, Parvaneh N (2011). Impact of delayed diagnosis in children with primary antibody deficiencies. J Microbiol Immunol Infect.

[10] Maarschalk-Ellerbroek LJ, Hoepelman AIM, Van Montfrans JM, Ellerbroek PM (2012). The spectrum of disease manifestations in patients with common variable immunodeficiency disorders and partial antibody deficiency in a university hospital. J Clin Immunol.

[11] El-Helou SM, Biegner AK, Bode S, Ehl SR, Heeg M, Maccari ME (2019). The German national registry of primary immunodeficiencies (2012-2017). Front Immunol.

[12] Odnoletkova I, Kindle G, Quinti I, Grimbacher B, Knerr V, Gathmann B (2018). The burden of common variable immunodeficiency disorders: a retrospective analysis of the European Society for Immunodeficiency (ESID) registry data. Orphanet J Rare Dis.

[13] Aghamohammadi A, Pouladi N, Parvaneh N, Yeganeh M, Movahedi M, Gharagolou M (2007). Mortality and morbidity in common variable immunodeficiency. J Trop Pediatr.

[14] Barlogis V, Mahlaoui N, Auquier P, Pellier I, Fouyssac F, Vercasson C (2017). Physical health conditions and quality of life in adults with primary immunodeficiency diagnosed during childhood: a French Reference Center for PIDs (CEREDIH) study. J Allergy Clin Immunol.

[15] Modell V, Gee B, Lewis DB, Orange JS, Roifman CM, Routes JM (2011). Global study of primary immunodeficiency diseases (PI)-diagnosis, treatment, and economic impact: an updated report from the Jeffrey Modell Foundation. Immunol Res.

[16] Messelink MA, Berbers RM, van Montfrans JM, Ellerbroek PM, Gladiator A, Welsing PM, Leavis H (2023). Development of a primary care screening algorithm for the early detection of patients at risk of primary antibody deficiency. Allergy, Asthma Clin Immunol.

[17] Smeets HM, Kortekaas MF, Rutten FH, Bots ML, van der Kraan W, Daggelders G (2018). Routine primary care data for scientific research, quality of care programs and educational purposes: the Julius General Practitioners’ Network (JGPN). BMC Health Serv Res.

[18] Cunningham-Rundles C, Sidi P, Estrella L, Doucette J (2004). Identifying undiagnosed primary immunodeficiency diseases in minority subjects by using computer sorting of diagnosis codes. J Allergy Clin Immunol.

[19] Brodszki N, Jönsson G, Skattum L, Truedsson L (2014). Primary immunodeficiency in infection-prone children in southern Sweden: occurrence, clinical characteristics and immunological findings. BMC Immunol.

[20] Cunningham-Rundles C (2012). The many faces of common variable immunodeficiency. Hematology Am Soc Hematol Educ Program.

[21] Wiesik-Szewczyk E, Jahnz-Rozyk K (2020). From infections to autoimmunity: diagnostic challenges in common variable immunodeficiency. World J Clin Cases.

[22] Song J, Lleo A, Yang GX, Zhang W, Bowlus CL, Gershwin ME (2018). Common variable immunodeficiency and liver involvement. Clin Rev Allergy Immunol.

[23] Bjelac JA, Yonkof JR, Fernandez J (2019). Differing performance of the warning signs for immunodeficiency in the diagnosis of pediatric versus adult patients in a two-center tertiary referral population. J Clin Immunol.

[24] McCusker C, Upton J, Warrington R (2018). Primary immunodeficiency. Allergy Asthma. Clin Immunol.

[25] Jolles S, Borrell R, Zouwail S, Heaps A, Sharp H, Moody M (2014). Calculated globulin (CG) as a screening test for antibody deficiency. Clin Exp Immunol.

[26] Ochs HDSE, Winkelstein JA (2004). Antibody deficiencies. Immunologic disorders in infants and children.

[27] Tangye SG, Al-Herz W, Bousfiha A, Chatila T, Cunningham-Rundles C, Etzioni A (2020). Human inborn errors of immunity: 2019 update on the classification from the International Union of Immunological Societies Expert Committee. J Clin Immunol.

[28] Bousfiha A, Jeddane L, Picard C, Al-Herz W, Ailal F, Chatila T (2020). Human inborn errors of immunity: 2019 update of the IUIS phenotypical classification. J Clin Immunol.

[29] Rider NL, Coffey M, Kurian A, Quinn J, Orange JS, Modell V (2023). A validated artificial intelligence-based pipeline for population-wide primary immunodeficiency screening. J Allergy Clin Immunol.

[30] Luca L, Beuvon C, Puyade M, Roblot P, Martin M (2021). Selective IgA deficiency. Rev Med Interne.

[31] Cunningham-Rundles C (2001). Common variable immunodeficiency. Curr Allergy Asthma Rep.

[32] Bevolkingsonderzoek Borstkanker Facsheet 2021 Bilthoven: Rijksinstituut voorVolksgezondheid en Milieu; 2022 [updated 02-2022. Available from: https://www.rivm.nl/documenten/factsheet-borstkanker.

[33] Bevolkingsonderzoek Darmkanker Factsheet 2021 Bilthoven: Rijksinstituut voorVolksgezondheid en Milieu; 2022 [updated 02-2022. Available from:https://www.rivm.nl/sites/default/files/2022-02/22400658_013665_FS%20Bevolkingsonderzoek%20Darmkanker%202021_V3_TG.pdf.

[34] Bevolkingsonderzoek Baarmoederhalskanker Factsheet 2021 Bilthoven:Rijksinstituut voor Volksgezondheid en Milieu; 2022 [updated 02-2022. Availablefrom:https://www.rivm.nl/documenten/factsheet-baarmoederhalskanker.

[35] Modell V, Orange JS, Quinn J, Modell F (2018). Global report on primary immunodeficiencies: 2018 update from the Jeffrey Modell Centers Network on disease classification, regional trends, treatment modalities, and physician reported outcomes. Immunol Res.

[36] Sadeghi B, Abolhassani H, Naseri A, Rezaei N, Aghamohammadi A (2015). Economic burden of common variable immunodeficiency: annual cost of disease. Expert Rev Clin Immunol.

[37] Elsink K, van Montfrans JM, van Gijn ME, Blom M, van Hagen PM, Kuijpers TW (2020). Cost and impact of early diagnosis in primary immunodeficiency disease: a literature review. Clin Immunol.

[38] Rider NL, Cahill G, Motazedi T, Wei L, Kurian A, Noroski LM (2021). PI Prob: a risk prediction and clinical guidance system for evaluating patients with recurrent infections. PLoS One.

[39] Rider NL, Miao D, Dodds M, Modell V, Modell F, Quinn J (2019). Calculation of a primary immunodeficiency “risk vital sign” via population-wide analysis of claims data to aid in clinical decision support. Front Pediatr.

[40] Mayampurath A, Ajith A, Anderson-Smits C, Chang SC, Brouwer E, Johnson J (2022). Early diagnosis of primary immunodeficiency disease using clinical data and machine learning. J Allergy Clin Immunol Pract.

[41] Basílio N, Ramos C, Figueira S, Pinto D (2016). Worldwide usage of International Classification of Primary Care. Revista Brasileira de Medicina de Família e Comunidade.

[42] Lackey AE, Ahmad F, X-linked agammaglobulinemia. (2022). StatPearls [Internet].

[43] Blom M, Bredius RGM, van der Burg M. Future perspectives of newborn screening for inborn errors of immunity. Int J Neonatal. Screen. 2021;7(4) 10.3390/ijns7040074 PMC8628921 34842618

[44] Orange JS, Ballow M, Stiehm ER, Ballas ZK, Chinen J, De La Morena M (2012). Use and interpretation of diagnostic vaccination in primary immunodeficiency: a working group report of the Basic and Clinical Immunology Interest Section of the American Academy of Allergy, Asthma & Immunology. J Allergy Clin Immunol.

[45] L. Hakkaart-van Roijen NvdL, C. Bouwmans, T. Kanters, S. Swan Tan. (2015). Kostenhandleiding: Methodologie van kostenonderzoek en referentieprijzen voor economische evaluaties in de gezondheidszorg.

[46] Centraal Bureau voor de Statistiek (CBS) / Statistics Netherlands. Consumer Price Index [updated 14-03-2023. Available from: https://opendata.cbs.nl/statline/#/CBS/nl/dataset/83131NED/table?ts=1679511519388.

[47] Zorginstituut Nederland. Farmacotherapeutisch Kompas. [Available from: www.farmacotherapeutischkompas.nl.

