# Supplementary material for: Clinical Validation of a Primary Antibody Deficiency Screening Algorithm for Primary Care
Source: J Clin Immunol. 2023 Sep 16;43(8):2022–32. doi: 10.1007/s10875-023-01575-8 (PMC10660978; doi:10.1007/s10875-023-01575-8)
Supplement: Supplementary file 1 — (DOCX 181 kb) [file 10875_2023_1575_MOESM1_ESM.docx]

# Online resources

**Table S1** Screening algorithm for early detection of primary antibody deficiencies in a primary care setting (original version)

| **ANTIBIOTIC PRESCRIPTIONS** Total score in the 4 years before the censoring date was calculated. If a patient was enrolled in the GP clinic fewer than 4 years, this was corrected for with the formula: (total number of prescriptions*4)/(number of days enrolled/365). Prescriptions before the age of 6 were not taken into account. | | |
| --- | --- | --- |
| **ATC-code** | **Description** | **Score per prescription** |
| J01AA02 | Doxycycline | 2 |
| J01CA04 | Amoxicillin | 2 |
| J01CF05 | Flucloxacillin | 1 |
| J01CR02 | Amoxicillin / clavulanic acid | 2 |
| J01EE01 | Cotrimoxazole | 2 |
| J01FA01 | Erythromycin | 2 |
| J01FA09 | Clarithromycin | 2 |
| J01FA10 | Azithromycin | 2 |
| J01MA02 | Ciprofloxacin | 1 |
| J01MA12 | Levofloxacin | 2 |
| J01MA14 | Moxifloxacin | 2 |
| P01AB01 | Metronidazole | 1 |
| J01CE05 | Pheneticillin | 2 |
| J01CE02 | Phenoxymethylpenicillin | 2 |
| J01DB01 | Cefalexin | 2 |
| J01DC04 | Cefaclor | 2 |
| J01DD14 | Ceftibuten | 2 |
| J01DC02 | Cefuroxime axetil | 2 |
| S02CA03 | Hydrocortisone / colistin / bacitracin ear suspension | 0.5 |
| S02AA16 | Ofloxacin ear suspension | 0.5 |
| **RESPIRATORY TRACT INFECTIONS**  Score was attributed if the ICPC code was registered in the 10 years before the censoring date. | | |
| **ICPC code** | **Description** | **Score for presence of code** |
| H01 | Ear pain | 1 |
| H04 | Discharge from ear | 1 |
| H71 | Acute otitis media / myringitis | 2 |
| H72 | Otitis media with effusion | 2 |
| H74 | Chronic otitis media / other ear infections | 1 |
| H74.01 | Chronic otitis media | 1 |
| H74.02 | Mastoiditis | 2 |
| R05 | Coughing | 1 |
| R07 | Sneezing / nasal congestion / running nose | 0.5 |
| R09 | Symptoms/complaints sinuses | 1 |
| R73 | Furuncle / abscess nose | 2 |
| R74 | Acute upper respiratory tract infection | 2 |
| R74.01 | Common cold | 1 |
| R96 | Asthma | 2 |
| R90 | Hypertrophy / chronic infection tonsils / adenoid | 2 |
| R72 | Streptococcal pharyngitis / scarlet fever | 1 |
| R72.01 | Streptococcal pharyngitis | 1 |
| R72.02 | Scarlet fever | 1 |
| R74.02 | Acute pharyngitis | 1 |
| R75 | Acute / chronic rhinosinusitis | 2 |
| R75.01 | Acute rhinosinusitis | 2 |
| R75.02 | Chronic rhinosinusitis | 2 |
| R76 | Acute tonsillitis / peritonsillar abscess | 2 |
| R76.01 | Acute tonsillitis | 2 |
| R76.02 | Peritonsillar abscess | 1 |
| R77 | Acute laryngitis / tracheitis | 2 |
| R77.01 | Subglottic laryngitis / pseudo croup | 1 |
| R77.02 | Acute epiglottitis | 1 |
| R78 | Acute bronchitis / bronchiolitis | 2 |
| R81 | Pneumonia | 3 |
| R91 | Chronic bronchitis / bronchiectasis | 1 |
| R91.01 | Chronic bronchitis | 1 |
| R91.02 | Bronchiectasis | 4 |
| **GASTRO-INTESTINAL COMPLAINTS**  Score was attributed if the ICPC code is registered in the 10 years before the censoring date. | | |
| **ICPC code** | **Description** | **Score for presence of code** |
| D11 | Diarrhoea | 2 |
| D70 | Infectious diarrhoea, dysentery | 1 |
| D70.01 | Salmonella | 1 |
| D70.02 | Shigella-/Yersinia-/Campylobacter intestinal infection | 2 |
| D70.03 | Giardia | 2 |
| D73 | Presumed gastro-intestinal infection | 2 |
| D86 | Other peptic ulcer | 1 |
| D93 | Inflammatory Bowel Syndrome | 1 |
| D94 | Ulcerative colitis / Chronic enteritis | 1 |
| D94.01 | Ulcerative colitis | 1 |
| **OTHER INFECTIONS**  Score was attributed if the ICPC code was registered in the EHR at any time point before the censoring date. | | |
| **ICPC code** | **Description** | **Score for presence of code** |
| L70.01 | Osteomyelitis | 1 |
| L70.02 | Septic arthritis | 1 |
| N71 | Meningitis / Encephalitis | 2 |
| N71.01 | Bacterial meningitis | 2 |
| N71.02 | Viral meningitis | 2 |
| N71.03 | Encephalitis | 2 |
| N71.04 | Myelitis | 2 |
| **AUTO-IMMUNE SYMPTOMS**  Score was attributed if the ICPC code was registered in the EHR at any time point before the censoring date. | | |
| **ICPC code** | **Description** | **Score for presence of code** |
| B04 | Symptoms / complaints blood / blood forming organs | 1 |
| B81 | Pernicious / Folic acid anaemia | 1 |
| B82 | Other / Non-specified anaemia | 1 |
| B83 | Purpura / coagulation disorder / aberrant thrombocytes | 1 |
| B83.02 | Idiopathic thrombocytopenic purpura | 2 |
| L88 | Rheumatoid arthritis / related diseases | 1 |
| L88.01 | Rheumatoid arthritis | 2 |
| R83.02 | Sarcoidosis | 1 |
| S23.01 | Alopecia areata | 1 |
| S99.04 | Vitiligo | 1 |
| T86 | Hypothyroidism | 1 |
| T99.02 | Thyroiditis | 1 |
| T99.12 | Adrenal insufficiency | 1 |
| D94.02 | Crohn's Disease | 2 |
| D99.06 | Coeliac disease | 1 |
| N99 | Myasthenia Gravis | 1 |
| **MALIGNANCIES, LYMPHOPROLIFERATIVE- AND OTHER SYMPTOMS**  Score was attributed if the ICPC code was registered in the EHR at any time point before the censoring date. | | |
| **ICPC code** | **Description** | **Score** |
| D74 | Gastric cancer | 1 |
| B72 | Hodgkin's disease | 1 |
| B72.01 | Hodgkin's disease | 1 |
| B72.02 | Non-Hodgkin lymphoma | 2 |
| T08 | Weight loss | 1 |
| B87 | Splenomegaly | 2 |
| B02 | Lymphadenopathy | 1 |
| D96 | Hepatomegaly | 1 |
| T10 | Failure to thrive | 2 |
| A04 | Fatigue / weakness | 1 |
| B84 | Aberrant leukocytes | 0.5 |
| N94 | Other peripheral neuritis / neuropathy | 1 |
| **LABORATORY VALUES**  Score was attributed if a reduced lab value was registered in the EHR at any time point before the censoring date. | | |
| **Laboratory measure** | **Aberrant value** | **Score if aberrant value present** |
| IgG - total | < 7 g/L | 8 |
| IgG1 | < 4.9 g/L | 8 |
| IgG2 | < 1.5 g/L | 8 |
| IgG3 | < 0.2 g/L | 8 |
| IgG4 | < 0.08 g/L | 8 |
| IgM-total | < 0.4 g/L | 8 |
| IgA-total | < 0.7 g/L | 4 |
| Calculated globulin (total protein–albumin) | < 18g/L | 6 |
| **VISITS TO GENERAL PRACTITIONER** Both physical and electronic visits were taken into account. | | |
| **Description** | **Cut-off value** | **Score if visits ≥ cut-off value** |
| Visits to the GP clinic in the year before the censoring date. | ≥ 6 visits | 3 |
| **AMBIGUOUS CODES** For these codes, EHRs were screened up to the moment of the ambiguous diagnosis | | |
| **ICPC code** | **Description** | |
| B72 | Hodgkin’s lymphoma | |
| B72.01 | Hodgkin’s lymphoma | |
| B72.02 | Non-Hodgkin lymphoma | |
| A87.02 | Post-transplantation | |
| D74 | Gastric cancer | |
| D74 | Colon or rectal cancer | |
| **EXCLUSION CRITERIA** | | |
| **ICPC code / other** | **Description** | |
| B73 | Leukaemia | |
| B74.01 | Multiple Myeloma | |
| B90 | HIV-infection | |
| B90.01 | HIV seropositive without symptoms | |
| B90.02 | AIDS / AIDS-related complex | |
| P15.01 | Alcoholism | |
| P15.02 | Delirium tremens | |
| P15.03 | Wernicke–Korsakoff | |
| P19.03 | Addiction to hard drugs | |
| T06 | Anorexia nervosa / bulimia | |
| T06.01 | Anorexia nervosa | |
| T06.02 | Bulimia | |
| T99.01 | Immunodeficiency | |
| T99.10 | Cystic fibrosis | |
| Age | < 12 years or > 70 years | |

*AIDS*, acquired immune deficiency syndrome; *ATC*, anatomic therapeutic chemical; *EHR,* electronic health record; *GP*, general practitioner; *HIV*, human immunodeficiency virus; *ICPC*, International Classification of Primary Care; *Ig*, immunoglobulin

**Table S2** TRIPOD checklist for prediction model development and validation

| **Section/**  **Topic** | **Item** |  | **Checklist Item** | **Section(s)** |
| --- | --- | --- | --- | --- |
| **Title and abstract** | | | | |
| Title | 1 | D;V | Identify the study as developing and/or validating a multivariable prediction model, the target population, and the outcome to be predicted. | Title page |
| Abstract | 2 | D;V | Provide a summary of objectives, study design, setting, participants, sample size, predictors, outcome, statistical analysis, results, and conclusions. | Abstract |
| **Introduction** | | | | |
| Background and objectives | 3a | D;V | Explain the medical context (including whether diagnostic or prognostic) and rationale for developing or validating the multivariable prediction model, including references to existing models. | Introduction, Discussion |
|  | 3b | D;V | Specify the objectives, including whether the study describes the development or validation of the model or both. | Introduction |
| **Methods** | | | | |
| Source of data | 4a | D;V | Describe the study design or source of data (e.g., randomized trial, cohort, or registry data), separately for the development and validation data sets, if applicable. | Methods |
|  | 4b | D;V | Specify the key study dates, including start of accrual; end of accrual; and, if applicable, end of follow-up. | Methods |
| Participants | 5a | D;V | Specify key elements of the study setting (e.g., primary care, secondary care, general population) including number and location of centres. | Methods |
|  | 5b | D;V | Describe eligibility criteria for participants. | Methods |
|  | 5c | D;V | Give details of treatments received, if relevant. | NA |
| Outcome | 6a | D;V | Clearly define the outcome that is predicted by the prediction model, including how and when assessed. | Methods |
|  | 6b | D;V | Report any actions to blind assessment of the outcome to be predicted. | NA |
| Predictors | 7a | D;V | Clearly define all predictors used in developing or validating the multivariable prediction model, including how and when they were measured. | Methods, Tables S1 and E7 |
|  | 7b | D;V | Report any actions to blind assessment of predictors for the outcome and other predictors. | NA |
| Sample size | 8 | D;V | Explain how the study size was arrived at. | Methods |
| Missing data | 9 | D;V | Describe how missing data were handled (e.g., complete-case analysis, single imputation, multiple imputation) with details of any imputation method. | ^a^ |
| Statistical analysis methods | 10a | D | Describe how predictors were handled in the analyses. | Methods, Results, Tables S1 and S7 |
|  | 10b | D | Specify type of model, all model-building procedures (including any predictor selection), and method for internal validation. | Methods, Results, Tables S1 and S7 |
|  | 10c | V | For validation, describe how the predictions were calculated. | Methods |
|  | 10d | D;V | Specify all measures used to assess model performance and, if relevant, to compare multiple models. | Methods, Results |
|  | 10e | V | Describe any model updating (e.g., recalibration) arising from the validation, if done. | Methods, Results, Table S7 |
| Risk groups | 11 | D;V | Provide details on how risk groups were created, if done. | NA |
| Development vs validation | 12 | V | For validation, identify any differences from the development data in setting, eligibility criteria, outcome, and predictors. | Methods,^b^ |
| **Results** | | | | |
| Participants | 13a | D;V | Describe the flow of participants through the study, including the number of participants with and without the outcome and, if applicable, a summary of the follow-up time. A diagram may be helpful. | Fig. 1 |
|  | 13b | D;V | Describe the characteristics of the participants (basic demographics, clinical features, available predictors), including the number of participants with missing data for predictors and outcome. | Tables 2 and 3,^a^ |
|  | 13c | V | For validation, show a comparison with the development data of the distribution of important variables (demographics, predictors, and outcome). | ^b^ |
| Model development | 14a | D | Specify the number of participants and outcome events in each analysis. | Results, Fig. 1 |
|  | 14b | D | If done, report the unadjusted association between each candidate predictor and outcome. | NA |
| Model specification | 15a | D | Present the full prediction model to allow predictions for individuals (i.e., all regression coefficients, and model intercept or baseline survival at a given time point). | Tables S1 and S7 |
|  | 15b | D | Explain how to the use the prediction model. | Methods, Results, Tables S1 and S7 |
| Model performance | 16 | D;V | Report performance measures (with CIs) for the prediction model. | Results, Table 5 |
| Model updating | 17 | V | If done, report the results from any model updating (i.e., model specification, model performance). | Results, Table S7 |
| **Discussion** | | | | |
| Limitations | 18 | D;V | Discuss any limitations of the study (such as non-representative sample, few events per predictor, missing data). | Discussion |
| Interpretation | 19a | V | For validation, discuss the results with reference to performance in the development data, and any other validation data. | ^b^ |
|  | 19b | D;V | Give an overall interpretation of the results, considering objectives, limitations, results from similar studies, and other relevant evidence. | Discussion |
| Implications | 20 | D;V | Discuss the potential clinical use of the model and implications for future research. | Discussion |
| **Other information** | | | | |
| Supplementary information | 21 | D;V | Provide information about the availability of supplementary resources, such as study protocol, Web calculator, and data sets. | Availability of data |
| Funding | 22 | D;V | Give the source of funding and the role of the funders for the present study. | Title page |

*CI,* confidence interval; *D,* development; *GP*, general practitioner; *NA,* not applicable; *V,* validation
^a^As our algorithm was based on primary care electronic health record data on registered diagnostic codes, medication prescriptions, visits to the GP, and laboratory values, there were no missing values other than incomplete registration, which could not be verified. For the cost-effectiveness analysis, multiple imputation was performed to extrapolate the results from our current study implementing algorithm version 1, to estimate the impact of a proposed screening method using algorithm version 1 and subsequently version 3. This is described in the results section
^b^The original model (version 1) was developed based on aggregate subgroup primary care data and on expert opinion. Therefore no comparison on individual patient-level data could be made. Messelink M.A. BRM, van Montfrans J.M., Ellerbroek, P.M., Gladiator A., Welsing P.M.J., Leavis H.L. Development of a primary care screening algorithm for the early detection of patients at risk of primary antibody deficiency. *Allergy, Asthma & Clinical Immunology* 2023, 19(1):44.

**Table S3** Included patients with a PAD diagnosis

| Rank | Serum immunoglobulin results  (Presented as a range in g/L owing to privacy considerations) | Clinical presentation & treatment | Vaccine responses | Diagnosis |
| --- | --- | --- | --- | --- |
| 9 | **Total IgG 6–7**, **IgG1 4–5**, **IgG2 0.5–1.5**, IgG3 0.3–0.4, IgG4 0.2–0.3, IgM 0.4–0.5, IgA 1.0–2.0 | Recurrent RTIs, treated with prophylactic AB and additional AB on demand for breakthrough infections.  Medical history: asthma | Borderline normal pneumococcal response in 2017 (75%) | Isolated IgG subclass deficiency |
| 42 | Total IgG 8–9, IgG1 7–8, IgG2 2–3, **IgG3 0.1–0.2**, IgG4 0.3–0.4, IgM 0.9–1.0, IgA 2–3 | Recurrent RTIs, treated with prophylactic AB and additional AB on demand for breakthrough infections.  Medical history: asthma | Insufficient pneumococcal vaccine response (30%) | Isolated IgG Subclass deficiency |
| 98 | **Total IgG 6–7, IgG1 4–5,** IgG2 1–2, **IgG3 0.1–0.2**, IgG4 0.2–0.3, IgM 0.9–0.1, IgA 1–2 | Recurrent RTIs, otitis externa, recurrent fungal infections.  Medical history: asthma | Normal (100%) | Isolated IgG subclass deficiency |
| 191 | Total IgG 7–8, IgG1 5–6, **IgG2 1–2**, **IgG3 0.1–0.2**, IgG4 1–2, IgM 1–2, IgA 1–2.  At referral: **total IgG 6–7, IgG1 3.5–4.5, IgG2 0.5–1.5, IgG3 0.1–0.2** | Recurrent RTIs. Advice AB use on demand in case of RTIs, 5-yearly pneumococcal vaccine and IgG check in 1 year  Medical history: COPD | Borderline normal pneumococcal response (75%) | Isolated IgG subclass deficiency |
| 228 | Total IgG 9–10, IgG1 7–8, **IgG2 0.5–1.5**, IgG3 0.2–0.3, IgG4 0.2–0.3, IgM 0.7–0.8, IgA 2–3 | Chronic otitis media. Advice local treatment and AB on demand for other RTIs | Normal (90%) | Isolated IgG subclass deficiency |
| 279 | Total IgG 13–14, IgG1 10–11, IgG2 2–3, **IgG3 0.1–0.2**, IgG4 1–2, IgM 0.4–0.5, IgA 3–4 | Recurrent RTIs. Advice AB on demand. Medical history: asthma | Insufficient pneumococcal vaccine response (65%) | Isolated IgG subclass deficiency |
| 317 | **Total IgG 6–7, IgG1 3–4,** IgG2 2–3, IgG3 0.2–0.3, IgG4 0.1–0.2, **IgM 0.3–0.4**, IgA 1–2 | Recurrent RTIs. Treatment by discretion of GP.  Medical history: colitis | Insufficient pneumococcal vaccine response (50%) | Isolated IgG subclass deficiency |
| 528 | **Total IgG 4–5, IgG1 3–4, IgG2 0.5–1.5**, IgG3 0.2–0.3, IgG4 0.1–0.2, IgM 0.5–0.6, IgA 2–3 | Recurrent RTIs. Start maintenance AB, consider immunoglobulin replacement therapy in case of insufficient infection control.  Medical history: COPD | Insufficient pneumococcal vaccine response (20%) | Isolated IgG subclass deficiency |
| 657 | Total IgG 7–8, IgG1 5–6, IgG2 1.5–2.5, IgG3 0.2–0.3, **IgG4 < 0.02, IgM 0.1–0.2,** IgA 2–3 | Recurrent RTIs. Start prophylactic ABs.  Medical history: IBS | Insufficient pneumococcal vaccine response (35%) | Specific Antibody Deficiency |
| 791 | **Total IgG 6–7, IgG1 3.5–4.5, IgG2 0.5–1.5,** IgG3 0.4–0.5, IgG4 0.1–0.2, **IgM 0.3–0.4,** IgA 1–2 | AB on demand, monitoring of immunoglobulins  Medical history: COPD | Insufficient pneumococcal vaccine response (40%) | Isolated IgG subclass deficiency |

*AB,* antibiotics; *COPD,* chronic obstructive pulmonary disease; *IBS,* irritable bowel syndrome; *Ig,* immunoglobulin; *RTI,* respiratory tract infection
Immunoglobulins are shown as a range in gram/litre owing to privacy regulations. Reduced values are depicted in bold. Pneumococcal vaccine response is based on comparison of pneumococcal antibody response against different serotypes (before and) after pneumovax23 (PPV23) vaccination, adequate in case of rise in titres of above 1.0 ug/mL for ≥ 70% of serotypes tested.*[44]* Immunoglobulins were considered reduced when: IgM < 0.4(< 0.28 age 12–16 yrs), IgA < 0.7, IgG total < 7(< 5.2 age 12–16 yrs), IgG1 < 4.9 (< 3.7 age 12–16 yrs), IgG2 < 1.5 (< 1.06 age 12–18 yrs), IgG3 < 0.20 (< 0.18 age 12–18 yrs), IgG4< 0.08 (< 0.035 age 12–18 yrs), calculated globulin < 18

**Table S4** Non-referred patients who had an advice for referral

| Rank | Reason referral advice  (Immunoglobulins presented as a range owing to privacy considerations) | Reason non-referral | (In)valid  Non-referral |
| --- | --- | --- | --- |
| 3 | Top 10% | Referral not deemed relevant by GP, antibiotics are prescribed by dermatologist owing to rosacea, GI-complaints due to IBS and recent cholecystectomy | Valid |
| 6 | Top 10%, IgG2 < 0.2 g/l and IgG4 < 0.01 | Referral not deemed relevant by GP as patient has a COPD diagnosis | Invalid |
| 22 | Top 10% | Referral not deemed relevant by GP. Patient used maintenance AB for asthma, which has been terminated since half a year | Invalid |
| 35 | Top 10% | Referral declined by patient, too much effort | Invalid^a^ |
| 88 | IgG2 0.5–1.5 g/l | Referral not deemed relevant by GP as patient only has recurrent urinary tract infections, but no other infections | Valid |
| 128 | IgG3 0.1–0.2 g/l | Referral declined by patient, too much effort | Invalid^a^ |
| 134 | IgM 0.3–0.4 g/l | Not deemed relevant by GP as IgM was previously reduced when measured by another specialist, recurrent RTIs present | Invalid |
| 146 | IgG2 0.5–1.5 g/l, IgG3 0.1–0.2 g/l | Referral not deemed relevant by GP as patient has about six infections a year, not deemed enough to label as recurrent infections | Invalid |
| 226 | Total IgG 5–6 g/l, IgM 0.3–0.4 g/l, IgG1 4–5 g/l, IgG2 0.5–1.5 g/l, IgG3 0.2–0.3 g/l | Referral declined by patient, too much effort | Invalid^a^ |
| 289 | IgM 0.3–0.4 g/l | Referral declined by patient, too much effort | Invalid^a^ |
| 317 | Total IgG 6–7 g/l, IgM 0.3–0.4 g/l, IgG1 3–4 g/l | Patient previously referred for recurrent infections, retrospectively PAD diagnosis can be made based on abnormal vaccine responses, hypogammaglobulinemia, and recurrent infections | Valid |
| 360 | IgG3 0.1–0.2 g/l | No referral made owing to change in GPs during study resulting in miscommunication. Possible future referral would be after study closure | Invalid |
| 430 | IgG3 < 0.03 g/l | Referral declined by patient, already too many hospital visits | Invalid^a^ |
| 458 | IgA 0.5–0.6 g/l | Referral not deemed relevant by GP as there are no ongoing recurrent infections and laboratory aberrances are mild | Valid |
| 652 | Total IgG 6–7 g/l, IgM 0.3–0.4 g/l, IgG2 0.5–1.5 g/l | Patient and GP together decided there was no indication for referral, as there were no ongoing recurrent infections | Valid |
| 1050 | IgG3 0.1–0.2 g/l, IgG4 < 0.04 g/l | Referral not deemed relevant by GP as patient does not have ongoing recurrent infections, currently only psoriasis complaints | Valid |

*AB,* antibiotics; *COPD*, chronic obstructive pulmonary disease; *GI*, gastro-intestinal, g/l: gram/litre, *GP*, general practitioner; *IBS*, irritable bowel syndrome; *Ig*, immunoglobulin; *PAD*, primary antibody deficiency
^a^Labelled as invalid as PAD cannot be excluded for these patients
Immunoglobulins are shown as a range in gram/litre owing to privacy regulations

**Table S5** Overview of referred patients in whom PAD could neither be confirmed nor excluded

| Rank | Reason referral advice (Immunoglobulins presented as a range owing to privacy considerations) | Outcome referral | Reason inconclusive diagnosis |
| --- | --- | --- | --- |
| 7 | Top 10% | Patient with recurrent infections and COPD diagnosis, for which prophylactic AB. Normal immunoglobulins. Vaccine responses were not assessed | Specific Antibody Deficiency cannot be excluded |
| 24 | Top 10% | Patient with recurrent infections and COPD diagnosis, for which prophylactic AB. Despite pneumococcal vaccination PVR23 response not assessed. Normal immunoglobulins | Specific Antibody Deficiency cannot be excluded |
| 77 | IgG1 4–5 g/l | Patient with recurrent infections, bronchiectasis, and COPD diagnosis, for which maintenance antibiotic therapy. Igs upon repeated measurement: IgG1 3.6 g/l, IgM 0.39 g/l. Normal vaccine responses | Isolated IgG subclass deficiency cannot be excluded owing to use of immunosuppressants |
| 107 | IgG2 0.5–1.5 g/l | Patient with recurrent infections and COPD diagnosis, for which maintenance antibiotic therapy. Igs upon repeated measurement IgG total 6.36 g/l, IgG2 1.31 g/l. Vaccine responses were not performed | Isolated IgG subclass deficiency cannot be excluded owing to prednisone use |
| 227 | IgG2 0.5–1.5 g/l, IgG4 < 0.03 g/l | Patient with recent pregnancy (discovered post-inclusion), therefore no vaccine responses were performed. Reduced Igs may be physiological. Follow up after pregnancy | PAD cannot be excluded, follow up after study closure |
| 803 | IgM 0.3-0.4 g/l | Late referral, vaccine responses have yet to be performed after study closure | Specific antibody deficiency cannot be excluded, follow up after study closure |

*AB*, antibiotics; *COPD*, chronic obstructive pulmonary disease; *Ig*, immunoglobulin; *PAD*, primary antibody deficiency

**Table S6** Comparison of EHRs that were used for recruitment of patients, and those that were within the top 400 but were not used for screening and recruitment of patients

|  | EHRs within top 400, used for patient recruitment | EHRs outside of top 400, used for patient recruitment | EHRs within top 400, *not* used for patient recruitment | *P* value |
| --- | --- | --- | --- | --- |
| Number of EHRs | 220 | 180 | 180 | NA |
| Excluded records during remote screening, number (%) | 38 (17.3%) | 19 (10.6%) | 18 (10.0%) | 0.051 |
| *Nephrotic syndrome / stage 3–4 liver cirrhosis* | *4 (1.8%)* | *2 (1.1%)* | *2 (1.1%)* | 1.000 |
| *Current chemotherapy* | *2 (0.9%)* | *1 (0.6%)* | *2 (1.1%)* |  |
| *Current pregnancy* | *2 (0.9%)* | *1 (0.6%)* | *1 (0.6%)* |  |
| *Short life expectancy/ deceased/moved away* | *4 (1.8%)* | *2 (1.1%)* | *3 (1.7%)* |  |
| *Severe alcohol/drug abuse* | *16 (7.3%)* | *10 (5.6%)* | *5 (2.8%)* |  |
| *Already treated by immunologist* | *2 (0.9%)* | *0 (0.0%)* | *2 (1.1%)* |  |
| *Anorexia/bulimia* | *1 (0.5%)* | *1 (0.6%)* | *2 (1.1%)* |  |
| *Post-transplantation* | *5 (2.3%)* | *1 (0.6%)* | *1 (0.6%)* |  |
| *Leukaemia / multiple myeloma* | *2 (0.9%)* | *1 (0.6%)* | *0 (0.0%)* |  |
| Excluded records during screening at GP, number (%) | 42 (19.1%) | 40 (22.2%) | *NA* | 0.440 |
| *Short life expectancy/ deceased/moved away* | *7 (3.2%)* | *2 (1.1%)* | *NA* | 0.962 |
| *Nephrotic syndrome* | *3 (1.4%)* | *2 (1.1%)* | *NA* |  |
| *Deemed unsuitable by GP* | *25 (11.4%)* | *32 (17.8%)* | *NA* |  |
| *Current pregnancy* | *1 (0.5%)* | *2 (1.1%)* | *NA* |  |
| *Multiple myeloma* | *1 (0.5%)* | *0 (0.0%)* | *NA* |  |
| *Already treated by immunologist* | *2 (0.9%)* | *1 (0.6%)* | *NA* |  |
| *Cystic fibrosis* | *1 (0.5%)* | *0 (0.0%)* | *NA* |  |
| *Stage 3–4 liver cirrhosis* | *1 (0.5%)* | *1 (0.6%)* | *NA* |  |
| Age, mean (SD) | 54.0 (14.0) | 50.1 (15.6) | 40.4 (16.3) | < 0.001 |
| Female, n(%) | 164 (74.5) | 130 (72.2) | 127 (70.6) | 0.667 |
| Score on algorithm 1, median (IQR) | 28.1 (24.0–35.8) | 18.3 (17.0–20.0) | 24.9 (21.0–27.9) | < 0.001 |
| Score antibiotics, median (IQR) | 12.5 (8.0–23.0) | 3.0 (2.0–6.0) | 16.1 (12.0–20.2) | < 0.001 |
| Score ICPC codes related to RTI, median (IQR) | 9.0 (7.0–12.0) | 9.0 (6.0–10.0) | 5.0 (3.0–7.0) | < 0.001 |
| Score ICPC codes related to GI-complaints, median (IQR) | 2.0 (0.0–2.0) | 2.0 (0.0–2.0) | 0.0 (0.0–2.0) | < 0.001 |
| Score ICPC codes related to other infections, median (IQR) | 0.0 (0.0–0.0) | 0.0 (0.0–0.0) | 0.0 (0.0–0.0) | 0.204 |
| Score ICPC codes related to auto-immune symptoms, median (IQR) | 0.0 (0.0–1.0) | 0.0 (0.0–0.0) | 0.0 (0.0–0.0) | 0.116 |
| Score ICPC codes related to lymphoproliferative symptoms, median (IQR) | 1.0 (0.0–1.0) | 1.0 (0.0–1.5) | 1.0 (0.0–1.0) | < 0.001 |
| Patients with a previously known reduced number of immunoglobulin levels in their EHR, number (%) | 2.0 (0.9%) | 0.0 (0.0%) | 0.0 (0.0%) | 0.335 |
| Score GP-visits in the year before the censoring date, median (IQR) | 3.0 (3.0–3.0) | 3.0 (3.0–3.0) | 3.0 (3.0–3.0) | < 0.001 |

*AB*, antibiotics; *EHR*, electronic health record; *GI*, gastro-intestinal; *GP*, general practitioner; *ICPC*, International Classification of Primary Care; *IQR*, interquartile range; *n*, number; *NA*, not applicable; *RTI*, respiratory tract infection; *SD*, standard deviation

For continuous data, an ANOVA (parametric) or Kruskall-Wallis (non-parametric) test was performed. For categorical outcomes Chi-square tests were performed, unless with small (< 5) expected cell frequencies, in which case Fisher’s exact test was performed

**Table S7** Screening algorithm for early detection of primary antibody deficiencies in a primary care setting, optimized based on principal component and subsequent Ridge regression analyses per dimension and per category

| **ANTIBIOTICS** Sum of prescriptions in the 4 years before the censoring date. If a patient was enrolled in the GP clinic fewer than 4 years, this was corrected for with the formula: (total number of prescriptions*4)/(number of days enrolled/365). Prescriptions before the age of 6 were not taken into account. | | **Coefficient Ridge regression analysis per category**  3.90 e^-05^ | **Weight for category**  1 |
| --- | --- | --- | --- |
| **ATC-code** | **Description** | **Coefficient Ridge regression analysis per dimension** | **Weight per dimension** |
|  | Dimension 1 (sum of prescriptions in 4 years) | 1.53 e^-04^ | 2 |
| J01CF05 | Flucloxacillin |  |  |
| J01CR02 | Amoxicillin / clavulanic acid |  |  |
| J01MA02 | Ciprofloxacin |  |  |
|  | Dimension 2 (sum of prescriptions in 4 years) | 8.24 e^-05^ | 1 |
| J01AA02 | Doxycycline |  |  |
| J01FA10 | Azithromycin |  |  |
|  | Dimension 3 (sum of prescriptions in 4 years) | -6.27 e^-06^ | 0.5 |
| J01CA04 | Amoxicillin |  |  |
| J01EE01 | Cotrimoxazole |  |  |
| S02CA03 | Hydrocortisone / colistin / bacitracin ear suspension |  |  |
|  | Dimension 4 (sum of prescriptions in 4 years) | -1.99 e^-04^ | 0.5 |
| J01FA09 | Clarithromycin |  |  |
| J01MA12 | Levofloxacin |  |  |
|  | Dimension 5 (sum of prescriptions in 4 years) | 2.55 e^-04^ | 2 |
| J01CE05 | Pheneticillin |  |  |
| J01DC02 | Cefuroxime axetil |  |  |
| J01FA01 | Erythromycin |  |  |
|  | Dimension 6 (sum of prescriptions in 4 years) |  |  |
| J01MA14 | Moxifloxacin |  |  |
|  | Dimension 7 (sum of prescriptions in 4 years) | 3.83 e^-04^ | 2 |
| P01AB01 | Metronidazole |  |  |
| S02AA16 | Ofloxacin ear suspension |  |  |
|  | Dimension 8 (sum of prescriptions in 4 years) | -5.17 e^-04^ | 0.5 |
| J01CE02 | Phenoxymethylpenicillin |  |  |
|  | Dimension 9 (sum of prescriptions in 4 years) | NA* |  |
| J01DB01 | Cefalexin |  |  |
| J01DC04 | Cefaclor |  |  |
| J01DD14 | Ceftibuten |  |  |
| **RESPIRATORY TRACT INFECTIONS**  Score was attributed if the ICPC code was registered in the 10 years before the censoring date. | | **Coefficient Ridge regression analysis per category**  5.24 e^-05^ | **Weight for category**  1 |
| **ICPC code** | **Description** | **Coefficient Ridge regression analysis per dimension** | **Weight per dimension** |
|  | Dimension 1 (1 point if 1 ICPC, 2 points if ≥ 2 ICPCs) | 4.56 e^-05^ | 1 |
| H01 | Ear pain |  |  |
| H71 | Acute otitis media / myringitis |  |  |
| H74 | Chronic otitis media / other ear infections |  |  |
| R74 | Acute upper respiratory tract infection |  |  |
|  | Dimension 2 (1 point if 1 ICPC, 2 points if ≥ 2 ICPCs) | -2.73 e^-04^ | 0.5 |
| R75 | Acute / chronic rhinosinusitis |  |  |
| R75.01 | Acute rhinosinusitis |  |  |
| R75.02 | Chronic rhinosinusitis |  |  |
| R05 | Coughing |  |  |
|  | Dimension 3 (1 point if present) | -7.66 e^-04^ | 0.5 |
| R76 | Acute tonsillitis / peritonsillar abscess |  |  |
|  | Dimension 4 (1 point if ≥ 1 present) | 1.47 e^-03^ | 3 |
| R72.01 | Streptococcal pharyngitis |  |  |
| R76.02 | Peritonsillar abscess |  |  |
| R72 | Streptococcal pharyngitis / scarlet fever |  |  |
| R72.02 | Scarlet fever |  |  |
|  | Dimension 5 (1 point if ≥ 1 present) | 5.03 e^-04^ | 2 |
| H04 | Discharge from ear |  |  |
| H72 | Otitis media with effusion |  |  |
| H74.01 | Chronic otitis media |  |  |
|  | Dimension 6 (1 point if ≥ 1 present) | 9.48 e^-05^ | 1 |
| R76.01 | Acute tonsillitis |  |  |
| R96 | Asthma |  |  |
| R90 | Hypertrophy / chronic infection tonsils / adenoid |  |  |
|  | Dimension 7 (1 point if ≥ 1 present) | 2.99 e^-04^ | 2 |
| R81 | Pneumonia |  |  |
| R78 | Acute bronchitis / bronchiolitis |  |  |
| R91 | Chronic bronchitis / bronchiectasis |  |  |
| R91.01 | Chronic bronchitis |  |  |
|  | Dimension 8 (1 point if present) | -9.91 e^-05^ | 0.5 |
| R74.01 | Common cold |  |  |
|  | Dimension 9 (1 point if ≥ 1 present) | 8.87 e^-04^ | 2 |
| R09 | Symptoms/complaints sinuses |  |  |
| R73 | Furuncle / abscess nose |  |  |
| R77.01 | Subglottic laryngitis / pseudo croup |  |  |
|  | Dimension 10 (1 point if present) | -7.48 e^-04^ | 0.5 |
| R91.02 | Bronchiectasis |  |  |
|  | Dimension 11 (1 point if ≥ 1 present) | 6.29 e^-04^ | 2 |
| R07 | Sneezing / nasal congestion / running nose |  |  |
| R77 | Acute laryngitis / tracheitis |  |  |
|  | Dimension 12 (1 point if ≥ 1 present) | -7.70 e^-04^ | 0.5 |
| H74.02 | Mastoiditis |  |  |
| R74.02 | Acute pharyngitis |  |  |
|  | Dimension 13 (1 point if present)  Code did not occur in data set of included PAD/unlikely PAD patients, categorized on clinical relevance | NA* | 2 |
| R77.02 | Acute epiglottitis |  |  |
| **GASTRO-INTESTINAL COMPLAINTS**  Score was attributed if the ICPC code is registered in the 10 years before the censoring date. | | **Coefficient Ridge regression analysis per category** 1.72 e^-05^ | **Weight for category**  1 |
| **ICPC code** | **Description** | **Coefficient Ridge regression analysis per dimension** | **Weight per dimension** |
|  | Dimension 1 (1 point if ≥ 1 present) | 3.01 e^-04^ | 2 |
| D94 | Ulcerative colitis / Chronic enteritis |  |  |
| D94.01 | Ulcerative colitis |  |  |
| D11 | Diarrhoea |  |  |
|  | Dimension 2 (1 point if ≥ 1 present) | -7.19 e^-04^ | 0.5 |
| D86 | Other peptic ulcer |  |  |
| D70.01 | Salmonella |  |  |
|  | Dimension 3 (1 point if ≥ 1 present) | -6.84 e^-04^ | 0.5 |
| D73 | Presumed gastro-intestinal infection |  |  |
| D70 | Infectious diarrhoea, dysentery |  |  |
|  | Dimension 4 (1 point if ≥ 1 present) | 1.50 e^-05^ | 1 |
| D93 | Inflammatory Bowel Syndrome |  |  |
| D70.03 | Giardia |  |  |
|  | Dimension 5 (1 point if present) | -7.14 e^-04^ | 0.5 |
| D70.02 | Shigella-/Yersinia-/Campylobacter intestinal infection |  |  |
| **OTHER INFECTIONS**  Score was attributed if the ICPC code was registered in the EHR at any time point before the censoring date. | | **Coefficient Ridge regression analysis per category**  NA* | **Weight for category**  3 |
| **ICPC code** | **Description** | **Coefficient Ridge regression analysis per dimension** | **Weight per dimension** |
|  | Dimension 1 (1 point if ≥ 1 present) | NA* | 1 |
| L70.01 | Osteomyelitis |  |  |
| L70.02 | Septic arthritis |  |  |
|  | Dimension 2 (1 point if ≥ 1 present) | NA* | 1 |
| N71 | Meningitis / Encephalitis |  |  |
| N71.01 | Bacterial meningitis |  |  |
| N71.02 | Viral meningitis |  |  |
| N71.03 | Encephalitis |  |  |
| N71.04 | Myelitis |  |  |
| **AUTO-IMMUNE SYMPTOMS**  Score was attributed if the ICPC code was registered in the EHR at any time point before the censoring date. | | **Coefficient Ridge regression analysis per category**  5.77 e^-04^ | **Weight for category**  3 |
| **ICPC code** | **Description** | **Coefficient Ridge regression analysis per dimension** | **Weight per dimension** |
|  | Dimension 1 (1 point if present) | -8.82 e^-04^ | 0.5 |
| L88 | Rheumatoid arthritis / related diseases |  |  |
|  | Dimension 2 (1 point if ≥ 1 present) | NA* | 2 |
| B83.02 | Idiopathic thrombocytopenic purpura |  |  |
| T99.02 | Thyroiditis |  |  |
|  | Dimension 3 (1 point if ≥ 1 present) | 1.09 e^-03^ | 1 |
| B81 | Pernicious / Folic acid anaemia |  |  |
| B82 | Other / Non-specified anaemia |  |  |
| T86 | Hypothyroidism |  |  |
|  | Dimension 4 (1 point if ≥ 1 present) | 1.04 e^-03^ | 1 |
| L88.01 | Rheumatoid arthritis |  |  |
| B83 | Purpura / coagulation disorder / aberrant thrombocytes |  |  |
| D99.06 | Coeliac disease |  |  |
|  | Dimension 5 (1 point if ≥ 1 present) | 1.04 e^-03^ | 1 |
| B04 | Symptoms / complaints blood / blood forming organs |  |  |
| S23.01 | Alopecia areata |  |  |
|  | Dimension 6 (1 point if present) | -8.47 e^-04^ | 0.5 |
| D94.02 | Crohn's Disease |  |  |
|  | Dimension 7 (1 point if present) | -8.59 e^-04^ | 0.5 |
| S99.04 | Vitiligo |  |  |
| N99 | Myasthenia Gravis |  |  |
|  | Dimension 8 (1 point if present) | NA* | 1 |
| R83.02 | Sarcoidosis |  |  |
|  | Dimension 9 (1 point if present) | NA* | 0.5 |
| T99.12 | Adrenal insufficiency |  |  |
| **MALIGNANCIES, LYMPHOPROLIFERATIVE- AND OTHER SYMPTOMS**  Score was attributed if the ICPC code was registered in the EHR at any time point before the censoring date. | | **Coefficient Ridge regression analysis per category**  1.46 e^-04^ | **Weight for category**  2 |
| **ICPC code** | **Description** | **Coefficient Ridge regression analysis per dimension** | **Weight per dimension** |
|  | Dimension 1 (1 point if ≥ 1 present) | 6.63 e^-04^ | 2 |
| B72 | Hodgkin's disease |  |  |
| B72.01 | Hodgkin's disease |  |  |
| B72.02 | Non-Hodgkin lymphoma |  |  |
|  | Dimension 2 (1 point if ≥ 1 present) | NA* | 1 |
| T08 | Weight loss |  |  |
| B02 | Lymphadenopathy |  |  |
| A04 | Fatigue / weakness |  |  |
|  | Dimension 3 (1 point if ≥ 1 present) | -2.70 e^-05^ | 0.5 |
| B84 | Aberrant leukocytes |  |  |
| N94 | Other peripheral neuritis / neuropathy |  |  |
|  | Dimension 4 (1 point if ≥ 1 present) | NA* | 2 |
| B87 | Splenomegaly |  |  |
| D96 | Hepatomegaly |  |  |
|  | Dimension 5 (1 point if present) | NA* | 1 |
| T10 | Failure to thrive |  |  |
|  | Dimension 6 (1 point if present) |  | 1 |
| D74 | Gastric cancer |  |  |
| **LABORATORY VALUES**  Score was attributed if a reduced lab value was registered in the EHR at any time point before the censoring date. | | **Coefficient Ridge regression analysis per category** NA* | **Weight for category**  2 |
| **Reduced laboratory measure** | | **Coefficient Ridge regression analysis per dimension** | **Weight per dimension** |
| Dimension 1 (1 point if ≥ 1 present) | | NA* | 1 |
| IgG4 < 0.08 g/L | |  |  |
| IgM-total < 0.4 g/L | |  |  |
| IgA-total < 0.7 g/L | |  |  |
| Dimension 2 (1 point if ≥ 1 present) | | NA* | 2 |
| IgG–total < 7 g/L | |  |  |
| IgG1 < 4.9 g/L | |  |  |
| IgG2 < 1.5 g/L | |  |  |
| IgG3 < 0.2 g/L | |  |  |
| Calculated globulin (total protein–albumin) < 18g/L | |  |  |
| **VISITS TO GP** ≥ 6 visits to the GP clinic in the year before the censoring date. Both physical and electronic visits were taken into account. | | **Coefficient Ridge regression analysis per category**  2.81 e^-04^ | **Weight for category**  2 |
| **AMBIGUOUS CODES** For these codes, EHRs were screened up to the moment of the ambiguous diagnosis | | | |
| **ICPC code** | **Description** | | |
| B72 | Hodgkin’s lymphoma | | |
| B72.01 | Hodgkin’s lymphoma | | |
| B72.02 | Non-Hodgkin lymphoma | | |
| A87.02 | Post-transplantation | | |
| D74 | Gastric cancer | | |
| D74 | Colon or rectal cancer | | |
| **EXCLUSION CRITERIA** | | | |
| **ICPC code / other** | **Description** | | |
| B73 | Leukaemia | | |
| B74.01 | Multiple Myeloma | | |
| B90 | HIV-infection | | |
| B90.01 | HIV seropositive without symptoms | | |
| B90.02 | AIDS / AIDS-related complex | | |
| P15.01 | Alcoholism | | |
| P15.02 | Delirium tremens | | |
| P15.03 | Wernicke–Korsakoff | | |
| P19.03 | Addiction to hard drugs | | |
| T06 | Anorexia nervosa / bulimia | | |
| T06.01 | Anorexia nervosa | | |
| T06.02 | Bulimia | | |
| T99.01 | Immunodeficiency | | |
| T99.10 | Cystic fibrosis | | |
| Age | < 12 years or > 70 years | | |

*AIDS*, acquired immune deficiency syndrome; *ATC*, anatomic therapeutic chemical; *EHR*, electronic health record; *GP*, general practitioner; *HIV*, human immunodeficiency virus; *ICPC*, International Classification of Primary Care; *Ig*, immunoglobulin; *NA*; not applicable

Individual items were reduced to dimensions based on a principal component analysis using the data of 580 primary care patients. Subsequently, the weights per dimension (within a category) was determined based on the coefficients from a Ridge regression analysis for each category, using the data from 88 PAD/unlikely PAD patients who were included in the GP-PAD II study. Lastly, the weights per category were determined by performing an overarching Ridge regression analysis, in which the variables consisted of the scores per category of the algorithm (adjusted based on weights dimensions)

*If a prescription, ICPC code, or laboratory value did not occur in the data set of included PAD/unlikely PAD patients, Ridge regression analysis could not be performed. In this case, the dimension and weight per dimension were based on clinical relevance

**Table S8** Overview of estimated costs of the PAD screening algorithm when applying version 1 and subsequently version 3 of the algorithm to our study population of 61 172 patients

| Description | Estimated cost for one patient^a^ | Estimated number of patients to whom this step would apply | Total estimated cost |
| --- | --- | --- | --- |
| EHR screening, performed by doctor’s assistant, 10 minutes per EHR at a gross hourly rate of €14.07 | €2.34 | 296 | €694.12 |
| Blood withdrawal and serum analysis of immunoglobulins (IgA, IgM, IgG total, IgG1, IgG2, IgG3, IgG4, total protein, and albumin) | €191.55 | 149 | €28 540.66 |
| Two outpatient visits to an immunologist at an academic hospital^b^ [45, 46] | €398.26 | 46 | €18 319.96 |
| Laboratory testing including blood count, leukocyte differentiation, ferritin, HIV-test, and urine sediment | €56.09 | 46 | €2580.14 |
| Pneumococcal vaccination | €25.94 | 46 | €1193.24 |
| 2x pneumococcal vaccination response[47] | €27.36 | 46 | €1258.56 |
| Total | | | **€52 586.68** |

*EHR*, electronic health record; *HIV*, human immunodeficiency virus; *Ig*, immunoglobulin

^a^Based on expenses in current study or internal prices of the University Medical Centre Utrecht, unless references in the description specify otherwise. ^b^Costs based on estimation by the Institute for Medical Technology Assessment of the Erasmus Universiteit Rotterdam, corrected for inflation

**Figure S1** Overview of included patients versus total population


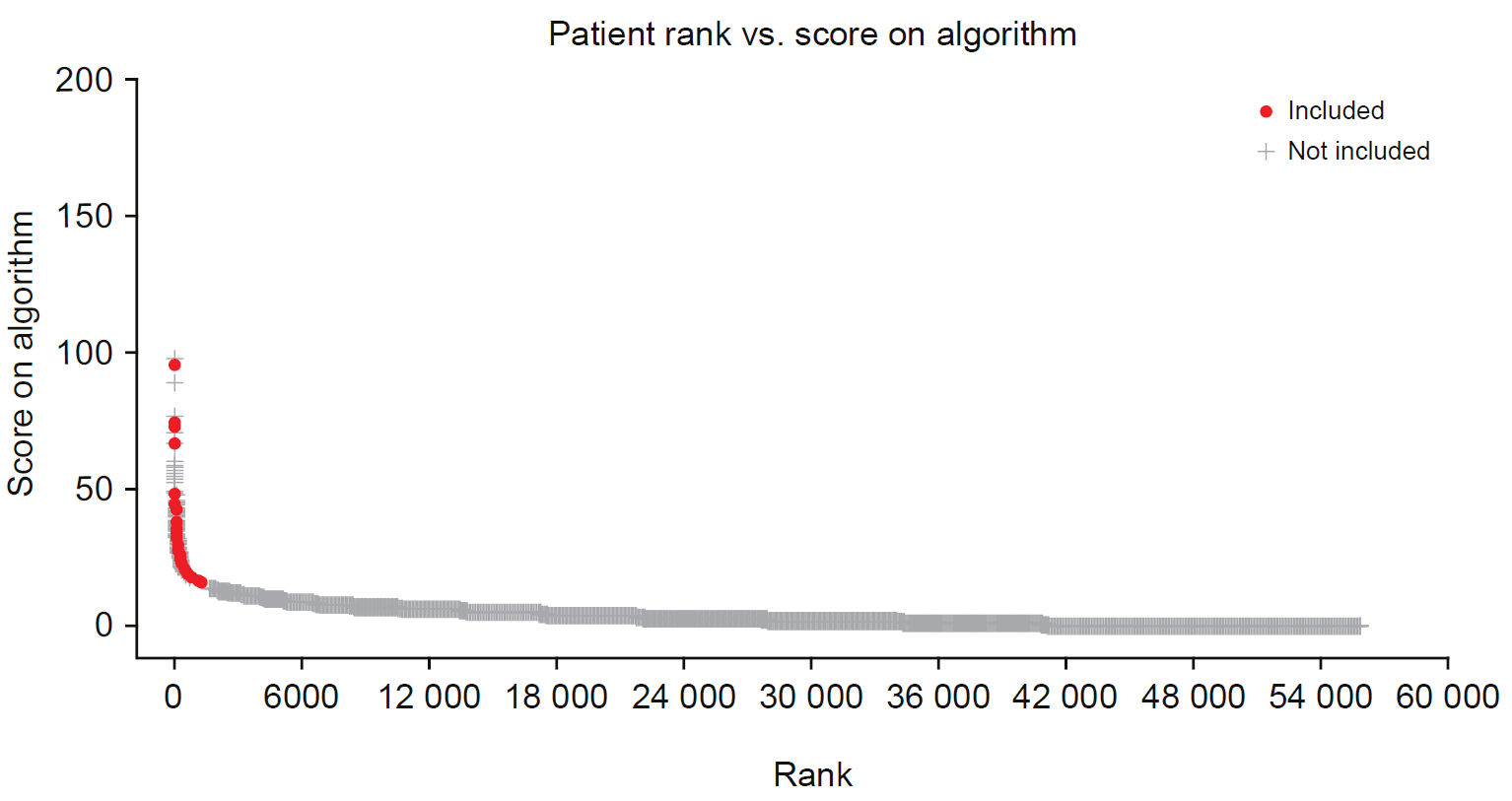


Total population based on 55 888 patients, i.e. initial study population of 61 172 minus 5284 patients excluded based on registered ICPC codes (International Classification of Primary Care). See also Figure 1 in main manuscript
